# Abastracts/Sommaires/Zusammenfassungen

**DOI:** 10.1155/S1463924680000223

**Published:** 1980


### Abstracts Sommaires Zusammenfassungen

### Page 57

**Automated systems for the clinical laboratory: the user's needs**

J. Bierens de Haan

The role of automated clinical instrumentation is discussed from the standpoint of the clinical chemist. Both users and instrument companies must cooperate to provide the equipment necessary to service health care requirements. The author discusses the requirements for cooperation, explains the reasons for the disparity of opinions and the need for communication. In addition the user's needs are outlined.

**Systèmes automatiques pour le laboratoire clinique: les besoins de l'utilisateur**

Le rôle de l'instrumentation automatique clinique est discuté du point de vue du chimiste clinique. Utilisateurs et fabricants d'instruments doivent coopérer pour réaliser l'éqiupment nécessaire aux besoins de contrôle de la santé. L'auteur discute les conditions d'une coopération, explique les raisons des différences d'opinion et la nécessité de communication. En plus les besoins des utilisateurs sont décrits.

**Automatische Systeme für das klinische Laboratorium: Die Bedurfnisse des Benützers**

Die Rolle der automatisierten klinischen Instrumentierung wird aus der Sicht des klinischen Chemikers diskutiert. Benützer und Instrumentenhersteller müssen zusammenarbeiten um die Geräte erhältlich zu machen, die von der Gesundheitsmedizin benötigt werden. Der Autor diskutiert die Notwendigkeit der Kooperation und erklärt die Grunde für Meinungsverschiedenheiten und die Bedürfnisse für die Kommunikation. Zusätzlich werden die Bedürfnisse des Benützers angeführt.

### Page 60

**A microprocessor controlled scanning polarograph for solution labile compounds**
R.E. Cooley, C.E. Stevenson and E.C. Rickard

An automatic, microprocessor controlled polarograph is described. The system utilises a solid sampler, designed in the authors laboratory, for the dissolution of powered samples. The sampler dissolves the sample just prior to analysis, thus making it of particular utility in the automatic analysis of solution labile compounds. Many of the problems found in other solid samplers have been eliminated by this design. An example of the precision obtained for the polarographic analysis of a cephalosporin antibiotic is given.

**Polargraphe controlé par microprocessuer pour substances labiles en solution**

Un polarographe automatique controlé par un microprocesseur est décrit. Le systèm utilise un preneur d'échantillons développé dans nos laboratoires pour la dissolution d'échantillons solides pulverisés. Le preneur d'échantillons dissout l'échantillon immédiatement avan l'analyse. Il est donc surtout utile pour l'analyse automatique de substances labiles en solution. La plupart des problèmes qu'on rencontre avec d'autres preneurs d'échantillons, ont pu être éliminés par cette construction. Un exemple est donné de la précision obtenue pour l'analyse polarographique de l'antibiotique Cephalosporine.

**Ein Mikroprozessor-gesteuerter Polarograph für lösungslabile Verbindungen**

Ein automatischer Mikroprozessor -gesteuerter Polarograph wird beschrieben. Das System verwendet einen in den Laboratorien des Autors entworfenen Probennehmer für feste Proben zur Auflösung der pulverförmigen Proben. Der Probennehmer löst die Probe unmittelbar vor der Analyse auf. Derart ist er besonders nützlich für automatische Analyse lösungslabiler Verbindungen. Viele der Probleme, die bei anderen Probennehmern für feste Proben autreten, konnten durch diese Konstruktion eliminiert werden. Ein Beispiel wird angegeben für die erreichte Präzision in der polarographischen Analyse eines Cephalosporin-Antibiotikums.

### Page 64

**An automatic collector for density gradients**
Rex Mottershead

An automatic device has been developed to collect nucleic acids in density gradients onto paper tape for subsequent estimation in a scintillation counter. This process is normally carried out by various manual methods which tend to be slow and tedious to perform. The device described has proved to be faster and more convenient than such methods.

**Un appareil collecteur automatique pour gradients de densité**

Un instrument a été développé pour la collection de gradients de densité d'acides nucléiques sur bandes de papier pour la determination dans un compteur à scintillations. Ce processus est normalement exécuté par différentes méthodes manuelles difficiles et lentes. L'instrument décrit s'avère plus rapide et confortable que les méthodes manuelles.

**Ein automatisches Auffanggerät für Dichtegradienten**

Es wurde ein automatisches Gerät entwickelt für das Auffangen nach Dichtegradienten von Nukleinsäuren auf Papierstreifen zur nachfolgenden Bestimmung in einem Szintillationszähler. Dieser Prozess wird normalerweise mit verschiedenen manuellen Methoden durchgeführt, die schwierig und langsam sind. Das beschriebene Gerät erwies sich als schneller und bequemer als solche Methoden.

### Page 66

**A compact automated microprocessor-based stopped-flow analyser**
Michael A Koupparis, Ken M. Walczak and Howard V. Malmstadt

A compact automated microprocessor-based stopped-flow analyser is described, the stepwise operations of the system are illustrated. The software flexibility allows various modes of operation and these are discussed. The system provides automatic aliquoting, mixing, transfer of solution to the photometer, and printout of results. Equilibrium and reaction-rate photometric methods for the determination of ascorbic acid, iron (III), phosphorus, creatinine and glucose are used to demonstrate typical precisions

**Un analysateur "stopped flow" compact et automatique, basé sur microprocesseur**

Un analysateur "stopped flow" compact et automatique, basé sur microprocesseur est décrit. Les opérations séquentielles sont illustrées. La flexibilité du software permet différents modes d'opération, qui sont discutés. Le système permet l'aliquotage automatique, le mélange, le transfert de solution au photomètre et l'impression des résultats. Des méthodes d'équilibre et de cinétique de réaction pour la détermination d'acide ascorbique, fer (III), phosphore, créatinine et glucose sont utilisées pour

**Kompakter, automatisierter Stopped-Flow Analysator auf Mikroprozessor-Basis**

Ein kompakter, automatisierter Stopped-Flow Analysator auf Mikroprozessor-Basis wird beschrieben und die einzelnen Schritte beim Betrieb des Systems werden erläutert. Die Flexibilität der Software erlaubt verschiedene Betriebsarten, die näher diskutiert werden. Das System führt automatisches Aliquotieren, Mischen und Transfer der Lösung zum Photometer durch und druckt die Resultate aus. Gleigewichts und reaktionskinetische Methoden der Photometrie werden benützt sur Bestimmung von of 0.1 - 0.7% and throughput capability of 100 - 200 samples per hour.

démontrer la précision typique de 0.1–0.7% et la capacité de traitement de 100–200 échantillons par heure.

Ascorbinsäure, Eisen (III), Phosphor, Kreatinin und Glucose um die typische Präzision von 0.1–0.7% aufzuzeigen bei einer Durchsatz-Kapazität von 100–200 Proben pro Stunde.

### Page 76

### Microprocessor-based monochromator controller

R. Dalle-Molle and J.D. Defreese

A simple microprocessor-based controller has been developed for the GCA/McPherson EU-700 monochromator. The microcomputer system consists of the Intel 8085 microprocessor, 8155 RAM –I/O – Timer, 8218 latch, and 2708 EPROM integrated circuits and controls the monochromator via a simple digital interface. The controller can function in a stand-alone mode, in which interaction with the operator is through a keyboard, or as an "intelligent" monochromator when put under the control of another computer. Features of the system include closed loop control of wavelength setting for improved accuracy and precision, bidirectional scan capability, and wavelength programming capability.

### Contrôleur de monochromateur basé sur microprocesseur

Un simple contrôleur basé sur microprocesseur a été développé pour le monochromateur GCA/McPherson EU–700. Le microordinateur consiste en un Intel 8085 microprocesseur, un 8155 RAM–I/O–Timer, un 8218 latch et un 2708 interface digital. Le contrôleur peut être utilisé par l'opérateur à l'aide d'un clavier ou comme monochromateur intélligent sous le contrôle d'un autre ordinateur. Le système inclut un contrôle "closed loop" de réglage de la longueur d'onde pour meilleure précision et reproductibilité, capacité de scan bidirectionelle et capacité de programmation de longueur d'onde.

### Monochromator-Steuerung mit Mikroprozessor

Für den GCA/McPherson EU–700 Monochromator wurde eine einfache Mikroprozessor-Steuerung entwickelt. Das Mikroprozessorsystem besteht aus dem Intel 8085 Mikroprocezzor, 8155 RAM–I/O–Timer, 8218 Latch, und 2708 Digital–Interface. Die Steuerung kann für sich alleine betrieben werden, wobei der Benützer über eine Tastatur Einfluss nimmt, oder aber die Steuerung funktioniert als "intelligenter" Monochromator unter Kontrolle eines übergeordneten Rechners. Die Eigenschaften des Systems umfassen gegengekoppelte Einstellung der Wellenlänge zwecks Erhöhung von Genauigkeit und Präzision, Vorschub in beiden Richtungen und Programmierbarkeit der Wellenlänge.

### Page 81

### Discrepancies in the calibration of reaction rate analysers

E.F. Legg, J.D. Cooper, M.R. Holland and J. Willis

This paper describes the causes of discrepancies between two LKB reaction rate analysers which produced markedly different results. A calibration procedure is proposed which will check the performance of most reaction rate analysers, and should result in better inter-laboratory agreement of enzyme assays.

### Calibrage d'un analysateur de cinétique de réaction

Ce travail décrit les causes des discrépences entre deux analysateurs de cinétique de réaction LKB, qui produisent des résultats nettement différents. Une procédure de calibrage est proposée, qui permettra de contrôler les performances de la plupart des analysateurs de cinétique de réaction et qui devrait résulter en une meilleure concordance des analyses d'enzymes entre les laboratoires.

### Kalibrierung von reaktionskinetischen Analysatoren

Diese Arbeit beschreibt die Ursachen für Diskrepanzen zwischen zwei LKB reaktions skinetischen Analysatoren, die deutlich unterschiedliche Resultate generierten. Es wird eine Kalibrierungs-Prozedur vorgeschlagen, die die Leistungsfähigkeit der meisten reaktionskinetischen Analysatoren überprüft und bei enzymatischen Analysen zu verbesserter Uebereinstimmung zwischen verschiedenen Laboratorien führen sollte.

### Page 85

### An evaluation of the Nova 2 ionised calcium instrument

J.A. Fyffe, A.S. Jenkins, H.N. Cohen, F.J. Dryburgh and M.D. Gardner

The Nova 2 ionised calcium analyser was evaluated. It was convenient to use and free from many of the problems associated with previous $Ca^{++}$analysers. The electrodes were found to be robust and did not require replacement during twelve months use. Interference from other ions was small, only $Mg^{++}$ and $Zn^{++}$having a significant effect. Between electrode differences for blood, plasma and serum are described.

### Evaluation de l'instrument Nova 2 pour calcium ionisé

L'analysateur Nova 2 pour calcium ionisé a été evalué. Il était pratique à utiliser et dépourvu des problèmes associés à d'autres analysateurs pour $Ca^{++}$. Les électrodes s'avéraient être solides et elles ont été utilisées pendant douze mois sans être remplacées. Les interférences dues à d'autres ions étaient insignfiantes, uniquement $Mg^{++}$ et $Zn^{++}$montraient un effet signifiactif. Les différences des électrodes pour l'analyse de sang, plasme et sérum, sont décrites.

### Eine Evaluation des Nova 2 Instruments für ionisiertes Kalzium

Der Nova 2 Analysator für ionisiertes Kalzium wurde einer Evaluation unterzogen. Er ist bequem zu benützen und befreit von vielen Problemen, die mit den vorgängigen $Ca^{++}$–Analysatoren verbunden waren. Die Elektroden stellten sich als robust heraus und mussten während 12–monatiger Benützung nicht ersetzt werden. Interferenzen von anderen Ionen waren gering, lediglich $Mg^{++}$ und $Zn^{++}$ zeigten eine signifikante Wirkung. Elektrodenunterscheide sind beschrieben für Blut, Plasma und Serum.

### Page 90

### Inter-laboratory precision and relative accuracy of mechanised and non-mechanised systems for blood gas analysis

Alain Feuillu, Michel Catheline and Andre Le Treut

An 18-month inter-laboratory quality control survey was undertaken to determine inter-laboratory precision and the relative accuracy of mechanised and manual systems of blood gas analysis. Mechanised apparatus for blood $pO_2$ measurement showed poor replicability and imprecision. Analysers involving single-point calibration also showed imprecision, a finding which supports a preference for two-point calibration.

### Contrôle de qualité de l'analyse du gaz sanguin: comparaison de systèmes automatiques et non-automatiques

Pendant une période de 18 mois, la qualité des analyses du gaz sanguin a été contrôlé mensuellement. Ces analyses ont été exécutées par 22 laboratoires d'hôpitaux français, certains utilisant des instruments automatiques et d'autres des instruments non-automatiques. Tandis que l'instrument automatique permet certainement un maniement simplifié par un personnel non-qualifié, les systèmes non-automatiques donnaient pour tous les paramètres mesurés des résultats plus précis.

### Qualitäts-Kontrolle bei der Blutgas-Analyse: automatische im Vergleich zu nicht-automatischen Systemen

Während einer 18-monatigen Periode wurden monatlich Qualitäts-beurteilungen von Blutgasanalysen gemacht, die von 22 französischen Spitallabors durchgefuhrt wurden unter Benützung von zum Teil automatischen and zum Teil nicht-automatischen Geräten. Während der automatische Apparat zweifelsohne vereinfachte Möglichkeiten der Probenhandhabung sogar zur Durchführung mit Hifspersonal bietet, ergaben die nicht-automatischen Systeme in Bezung auf Prazision für alle gemessenen Parameter bessere Resultate.

